# Transcranial direct current stimulation over left inferior frontal cortex improves speech fluency in adults who stutter

**DOI:** 10.1093/brain/awy011

**Published:** 2018-01-31

**Authors:** Jennifer Chesters, Riikka Möttönen, Kate E Watkins

**Affiliations:** 1 Department of Experimental Psychology, University of Oxford, Oxford, UK; 2 School of Psychology, University of Nottingham, Nottingham, UK

**Keywords:** stammering, speech disorder, non-invasive brain stimulation, randomized controlled trial

## Abstract

See Crinion (doi:10.1093/brain/awy075) for a scientific commentary on this article.

Stuttering is a neurodevelopmental condition affecting 5% of children, and persisting in 1% of adults. Promoting lasting fluency improvement in adults who stutter is a particular challenge. Novel interventions to improve outcomes are of value, therefore. Previous work in patients with acquired motor and language disorders reported enhanced benefits of behavioural therapies when paired with transcranial direct current stimulation. Here, we report the results of the first trial investigating whether transcranial direct current stimulation can improve speech fluency in adults who stutter. We predicted that applying anodal stimulation to the left inferior frontal cortex during speech production with temporary fluency inducers would result in longer-lasting fluency improvements. Thirty male adults who stutter completed a randomized, double-blind, controlled trial of anodal transcranial direct current stimulation over left inferior frontal cortex. Fifteen participants received 20 min of 1-mA stimulation on five consecutive days while speech fluency was temporarily induced using choral and metronome-timed speech. The other 15 participants received the same speech fluency intervention with sham stimulation. Speech fluency during reading and conversation was assessed at baseline, before and after the stimulation on each day of the 5-day intervention, and at 1 and 6 weeks after the end of the intervention. Anodal stimulation combined with speech fluency training significantly reduced the percentage of disfluent speech measured 1 week after the intervention compared with fluency intervention alone. At 6 weeks after the intervention, this improvement was maintained during reading but not during conversation. Outcome scores at both post-intervention time points on a clinical assessment tool (the Stuttering Severity Instrument, version 4) also showed significant improvement in the group receiving transcranial direct current stimulation compared with the sham group, in whom fluency was unchanged from baseline. We conclude that transcranial direct current stimulation combined with behavioural fluency intervention can improve fluency in adults who stutter. Transcranial direct current stimulation thereby offers a potentially useful adjunct to future speech therapy interventions for this population, for whom fluency therapy outcomes are currently limited.

## Teaser

Chesters *et al.* present the first randomized controlled trial of transcranial direct current stimulation (tDCS) as an adjunctive therapy for stuttering. Thirty adult males underwent a five-day intervention using choral and metronome-timed speech to induce fluency while receiving anodal or sham stimulation. tDCS reduced disfluency for at least 6 weeks.

## Introduction

Developmental stuttering is a neurodevelopmental condition disrupting the smooth flow of speech, resulting in characteristic speech disfluencies. Developmental stuttering has been associated with reduced educational and employment opportunities (Klein *et al.*, 2004; O'Brian *et al.*, 2011), social anxiety (Iverach *et al.*, 2009), and compromised quality of life (Craig *et al.*, 2009). Fluency therapies may use techniques for altering speech patterns to reduce overt stuttering (Boberg *et al.*, 1994; O'Brian *et al.*, 2003). However, fluency improvements do not persist without continued practice, and can be difficult to fully integrate into everyday speech. Furthermore, learning these new speech patterns can affect speech naturalness (Metz *et al.*, 1990; O'Brian *et al.*, 2003; Tasko *et al.*, 2007), which can reduce the acceptability of these approaches. There is a value, therefore, in developing novel interventions to improve therapy outcomes for adults who stutter.

Transcranial direct current stimulation (tDCS), a non-invasive brain stimulation method, may have potential to improve the outcomes of fluency interventions in people who stutter (Chesters *et al.*, 2017). TDCS involves application of a weak electrical current across the head via electrodes placed on the scalp, modulating the resting membrane potential of neurons in the underlying cortex. Anodal tDCS applied over motor cortex transiently enhances cortical excitability (Nitsche and Paulus, 2000). Critically, when paired with a task, the neuromodulatory effects of tDCS can improve motor learning (Nitsche *et al.*, 2003; Stagg and Nitsche, 2011), and this combination is understood to be an important factor in paradigms targeted at behavioural change (Stagg and Nitsche, 2011; Woods *et al.*, 2016). These improvements build and stabilize when applied in consecutive daily sessions (Reis *et al.*, 2009; Baker *et al.*, 2010). Increasingly, tDCS is being investigated as an adjunctive treatment for acquired disorders of motor, language and cognitive functions (Baker *et al.*, 2010; Marangolo *et al.*, 2011; Khedr *et al.*, 2013; Allman *et al.*, 2016; Mortensen *et al.*, 2016). For example, in a study treating upper limb motor function in stroke patients, tDCS was found to prolong the effects of 9 days of motor training for at least 3 months (Allman *et al.*, 2016). In post-stroke aphasic patients, 5 days of anodal tDCS over left inferior frontal cortex enhanced naming accuracy, which remained improved for at least 1 week post-intervention (Baker *et al.*, 2010). Here, we aimed to evaluate whether lasting fluency improvements could be obtained in a group of adults who stutter by combining tDCS with a 5-day behavioural fluency intervention.

People who stutter can experience near, or complete, fluency by changing the way speech is produced, for example by speaking with a different accent or in time with an external stimulus, such as a metronome or another speaker (so called ‘choral speech’). Altering the auditory feedback associated with speech production can also be effective; for example, feedback that is noisy, or altered in pitch or time (delayed) can result in almost complete fluency in some people (as portrayed in the film *The King's Speech*). It is important to note, however, that these forms of fluency induction, while successful at inducing almost complete fluency, are temporary and that disfluency returns typically once the inducer is removed. Although these fluency inducers are of little efficacy therapeutically, for our purposes their effectiveness in achieving immediate and close to complete fluency, with little impact on naturalness, was an important factor. We hypothesized that by applying tDCS while fluent speech was induced in people who stutter, we could facilitate the brain circuits supporting this fluent speech, promoting neuroplastic changes and thereby produce lasting fluency improvements.

The effectiveness of the temporary fluency inducers described above is consistent with theories that disfluency in people who stutter is caused by a problem in generating internal timing cues for motor control or sensorimotor integration or both (Alm, 2004; Max *et al.*, 2004; Watkins *et al.*, 2015). Brain imaging studies of adults who stutter confirm both structural and functional abnormalities in sensorimotor circuits involved in speech production. Since the first study showing reduced whiter matter integrity underlying left ventral sensorimotor cortex (Sommer *et al.*, 2002), numerous studies in children and adults who stutter have replicated this finding (Chang *et al.*, 2008, 2015; Watkins *et al.*, 2008; Kell *et al.*, 2009; Civier *et al.*, 2013; Connally *et al.*, 2014; for a review see Neef *et al.*, 2015). Functionally, there are differences in activation patterns in people who stutter that reflect both trait and state differences (see recent meta-analysis by Budde *et al.*, 2014). Of greatest relevance to our study, the left inferior frontal cortex is underactive during speaking in people who stutter (Wu *et al.*, 1995; Fox *et al.*, 1996; Neumann *et al.*, 2005; Watkins *et al.*, 2008; Kell *et al.*, 2009; Toyomura *et al.*, 2011). Furthermore, in our previous work in which both structural and functional data were obtained in the same participants, this region of functional under-activation was shown to overlie the white matter disruption (Watkins *et al.*, 2008), with a peak close to that identified in the meta-analysis as underactive for trait analysis by Budde and colleagues (Budde *et al.*, 2014). Following our feasibility study (Chesters *et al.*, 2017), the current study used a montage with the anode placed over the left inferior frontal cortex, covering also the ventral sensorimotor and premotor cortex. Based on the aforementioned functional and structural imaging findings, this cortical area appears to be a key region, which could benefit from increased activation to support fluent speech.

We recruited 30 male adults who stutter to a randomized double-blind controlled trial using tDCS in combination with a behavioural fluency intervention. The behavioural intervention involved temporarily inducing fluency using both choral speech and metronome-timed speech during overt reading, narrative and conversational speech tasks. We delivered 1 mA of anodal tDCS over the left inferior frontal cortex for 20 min per day in five consecutive daily sessions. Fluency was assessed 1 and 6 weeks after the 5-day intervention. We predicted that fluency intervention when combined with anodal tDCS would result in reduced disfluency (i.e. improved fluency), relative to the same fluency intervention with sham stimulation.

## Materials and methods

### Study design and participants

The study had a double-blind, sham-controlled, parallel-group design. A UK community sample of male adults aged 18–50 years, with at least a moderate stutter and with English as a first language, were recruited to participate. Exclusion criteria included any disorder of speech, language or communication other than developmental stuttering, sensory impairment, neurological or psychiatric illness, use of medications that act on the CNS, and any safety contra-indication for tDCS (e.g. personal or family history of seizures, taking medications or substances known to alter seizure threshold). A registered Speech and Language Therapist (J.C.) assessed stuttering, using the Stuttering Severity Instrument, version 4 (SSI-4, Riley, 2009), delivered the intervention, and completed all outcome and additional assessments, while blind to stimulation condition. The University of Oxford Central University Research Ethics Committee (MSD-IDREC-C2-2014-013) approved the study. Participants gave informed written consent to participate in the study, in accordance with the Declaration of Helsinki, and with the procedure approved by the committee. The trial was registered on ClinicalTrials.gov (NCT02288598).

### Randomization and masking

A researcher who was not involved in any aspect of the trial performed the randomization of participants into the sham and tDCS study arms using blocked randomization (Roberts and Torgerson, 1998). A block size of four was chosen generating six possible sequences, which were allocated at random. Allocation concealment was achieved by assigning a unique 5-digit code per participant. The code was used to deliver tDCS or sham stimulation using the ‘study mode’ on the stimulator (http://www.neurocaregroup.com/dc_stimulator_plus.html). Codes remained in sealed sequentially numbered opaque envelopes until allocation. The researcher who delivered the intervention, assessed the outcomes, and analysed the data, and the participants were masked to group assignment.

### Procedures

#### Transcranial direct current stimulation

**Figure 1:**
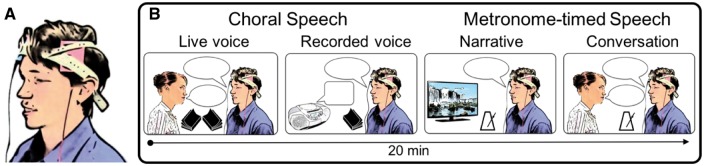
**TDCS montage and behavioural tasks used in intervention. **(**A**) Electrode placement montage used to apply tDCS. Anode (pink) was placed over left inferior frontal cortex, centred on position FC5 of the 10-10 EEG electrode placement system. Cathode (blue) was placed over the right supra-orbital ridge. (**B**) Choral speech (live voice and recorded voice) and metronome-timed speech (video narrative and conversation) tasks used in each daily intervention session. TDCS (1 mA) was applied concurrently with these tasks for 20 min in 15 male adults who stutter. Another 15 male adults who stutter received sham stimulation for the same period. Both researcher and participant were blind to the stimulation condition.

In the tDCS study arm, participants received 20 min of stimulation at 1 mA using 5 × 7 cm electrodes during the fluency intervention. These are frequently used tDCS parameters and were chosen as they have previously been effective in tDCS intervention studies (Marangolo *et al.*, 2011; Allman *et al.*, 2016). We also used these parameters in our own feasibility study (Chesters *et al.*, 2017). The anode was placed over left inferior frontal cortex (centred on FC5 according to the 10-10 EEG electrode placement system), and the cathode over the right supra-orbital ridge (Fig. 1A). This montage was tested in our feasibility study (Chesters *et al.*, 2017), and in a previous study of speech facilitation (Holland *et al.*, 2011). Electrode position FC5 is centred on Broca’s region, with the electrode extending posteriorly to cover ventral portions of premotor and primary motor cortex, where the representation of the articulators is located (Conant *et al.*, 2014). This electrode position covers the region of functional underactivity and white matter abnormality identified in people who stutter (Watkins *et al.*, 2008). A Neuroconn direct-current stimulator in ‘study mode’ was used to deliver tDCS, which enabled double blinding to stimulation condition. The electrodes were placed within saline soaked sponges and positioned on the scalp; the anode was placed in portrait orientation, and the cathode in landscape orientation. The same electrode placement was used in the sham stimulation study arm, during which the current was ramped up over 15 s, maintained for 15 s at 1 mA and ramped down over 15 s at the start of the session. For sham stimulation, the ‘study mode’ setting of the stimulator then delivered a small current pulse every 55 s (110 μA over 15 ms, with peak current lasting 3 ms) throughout the 20 min. These sham stimulation parameters delivered current at an ineffective dosage. The initial ramping of current ensured effective blinding of participants due to the same potentially adverse effects being felt at the start of stimulation (tingling or itching under the electrodes). The intermittent current pulse ensured effective blinding of both participant and researcher, as real impedance values were displayed on the stimulator in both tDCS and sham conditions.

#### Fluency intervention

A registered speech and language therapist delivered the fluency intervention in 20-min sessions on five consecutive days. We used behavioural techniques that induce temporary fluency. We chose these techniques for a maximal and immediate fluency induction because we wanted to be sure that application of tDCS would promote only the fluent state of speech and not the disfluent one. The behavioural techniques were choral speech, and metronome-timed speech (Kiefte *et al.*, 2008; Trajkovski *et al.*, 2009). Figure 1B illustrates the tasks used in each intervention session. Choral speech involved reading passages at a normal rate in unison first with a live voice and second with an audio-book recording. Metronome-timed speech involved speaking in time with an external audio metronome to produce spontaneous narratives of silent cartoon films followed by conversation with the researcher on randomly selected topics (e.g. a recent holiday). The metronome rate increased from 140 to 190 beats per minute across the 5 days. Participants were instructed to indicate if the rate exceeded a comfortable speaking rate at any time. In this case, the metronome was slowed to a comfortable rate, and maintained at this rate for the remaining days. Participants completed the tasks in a set order on each day of intervention, as shown in Fig. 1B, which was designed to form a hierarchy of difficulty. The hierarchy of difficulty was included to maintain engagement in the tasks whilst maximizing fluency, and to increase functional relevance. Tasks were modelled on Day 1 of the intervention, and participants were given feedback as needed in all sessions, to support them to maintain adequate task performance to induce fluency. Speech disfluency during reading and conversation was measured at baseline, before and after the intervention on each day, and at 1 week and 6 weeks post-intervention.

### Outcome measures

**Figure 2:**
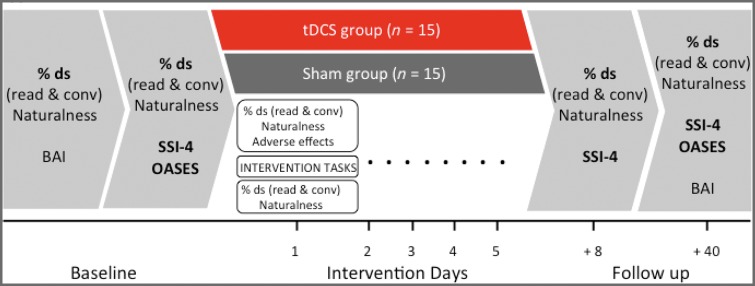
**Trial design. **Light grey boxes show baseline and outcome assessment time points. Measures used for the primary outcome (% ds = percentage of dysfluent syllables) and secondary outcomes (SSI-4, OASES) are shown in bold text. Additional measures used for matching group and monitoring adverse effects are shown in regular text. Exploratory measures taken pre-and post-intervention are shown in white boxes for Day 1 of the intervention only. These were repeated on each intervention day. BAI = Beck Anxiety Inventory.

Figure 2 summarizes all measures taken at each time point. The primary outcome measure for the trial was change from baseline proportion of stuttering in speech samples taken at 1 and 6 weeks post-intervention. A baseline percentage of disfluent syllables was estimated for two speech samples taken during reading and conversation on two separate days and averaged to give a stable estimate. The same measurement was taken post-intervention and the change from baseline at each time point calculated by subtracting the baseline from the post-intervention estimates. The primary outcome measure was overall change in fluency; we also analysed data from each task separately to explore whether the speaking situation produced different effects. Speech samples were collected for reading and conversation tasks at every outcome measurement time point, as well as immediately pre- and post-intervention on each intervention day. We analysed the first 2 min of each of these samples. This corresponds to a minimum of 200 syllables per sample and averaged at 560 for reading and 614 for conversation. Novel conversation topics and reading materials were used for every speech sample obtained for outcome measure assessment, and during the intervention. We defined disfluent syllables as those containing repetition or prolongation of a speech sound, or where a tense pause or ‘block’ occurred prior to a speech sound (i.e. core stuttering characteristics) as well as syllables in a repeated multi-syllabic word, a repeated phrase or phrase revision, a word fragment, or interjection (e.g. ‘um’, ‘err’).

We also measured how fluency was affected over the course of the intervention by including an additional assessment of the change in disfluency immediately after the intervention session on each of the 5 days of intervention.

One researcher completed all disfluency counts. Inter-rater reliability was measured by comparing all speech samples from two participants, selected at random, with counts independently completed by a second researcher. A strong intraclass correlation (ICC) was found for the inter-rater measurements (ICC = 0.94, *P* < 0.001), indicating a high level of reliability.

Secondary outcome measures included the SSI-4, which provides a standardized and norm-referenced index of disfluency, and the Overall Assessment of the Speaker’s Experience of Stuttering (OASES; Yaruss and Quesal, 2006), a self-assessment tool that measures the psycho-social impact of stuttering. The latter was used at baseline and at the 6-week post-intervention time point, to avoid violating retest reliability.

Speech naturalness was monitored across the trial as a reduction would be considered a possible adverse effect of stuttering intervention (Martin *et al.*, 1984; Onslow *et al.*, 1992, 1996; Teshima *et al.*, 2010). Speech naturalness was assessed for all speech samples using a nine-point Likert scale, with 1 representing highly natural sounding speech and 9 representing highly unnatural sounding speech. This is a commonly used scale for measuring speech naturalness in people who stutter (Ingham *et al.*, 1985; O'Brian *et al.*, 2003), and is included in the SSI-4 (Riley, 2009). The researcher completed this assessment during each session, and was blind to study arm. TDCS has been associated with mild and transient adverse effects. Therefore, we also monitored adverse effects related to receiving tDCS, such as an itching or tingling sensation at the electrode sites, using a questionnaire recommended from a previous review of tDCS adverse effect reporting (Brunoni *et al.*, 2011). As there is an increased prevalence of anxiety in developmental stuttering (Iverach *et al.*, 2009, 2011), all participants completed the Beck Anxiety Inventory (BAI; Beck *et al.*, 1988), to determine whether the groups differed with regard to anxiety symptoms.

### Statistical analysis

It was not possible to perform a power calculation based on previous trials of tDCS in developmental stuttering, as no studies prior to this one have been published. We made changes to the design based on the previous feasibility study (Chesters *et al.*, 2017), which precluded using that study as a basis for power analysis here (e.g. changes to the intervention, moving to multiple sessions, and a between-subjects design). Previous intervention studies using tDCS in patients with aphasia reported group differences of medium effect size [e.g. using a sample size of *n* = 10, (Baker *et al.*, 2010)]. With our sample size of 15 participants in each group, we had 80% power (with *P* < 0.05, one-tailed) to detect a large effect size (Cohen’s *d* > 0.9) for the main effect of stimulation.

Data were analysed according to the intention-to-treat principle. The distribution of the measurement of speech disfluency at baseline significantly deviated from normal, as is commonly seen in people who stutter (Jones *et al.*, 2006). To avoid the need for transformation (which is problematic for reporting confidence intervals in interpretable units; Bland and Altman, 1996), the trial outcomes were defined in terms of change from baseline, which was normally distributed.

The effect of tDCS on the primary outcome measure (change in % disfluent syllables from baseline) was assessed using a mixed-model analysis of variance (ANOVA), with a between-subjects factor of group (tDCS, sham) and two within-subjects factors: time post-intervention (1 week, 6 weeks) and speech task (reading, conversation). Further ANOVAs for the two groups separately were used to explore significant interactions. The effect of tDCS on the secondary outcome measure of (change from baseline in the SSI-4 score) was also assessed using a mixed-model ANOVA with the between-subject factor of group (tDCS, sham) and a within-subjects factor of time post-intervention (1 week, 6 weeks). As the other secondary outcome measure (change from baseline in OASES) was only acquired 6 weeks post-intervention, the effect of tDCS on this measure was assessed using an independent samples *t*-test between the two groups. For the additional analysis of the effects of tDCS during the 5-day intervention on speech fluency, we entered the change from baseline % disfluent syllables measured post-intervention on each day into a mixed-model ANOVA. Group (tDCS, sham) was the between-subjects factor and speech task (reading, conversation) and intervention day (one to five) were within-subjects factors. Mean adverse effects ratings for each session were entered into a mixed model ANOVA with group as the between-subjects factor (tDCS, sham) and intervention day as the within-subjects factor (session one to five). Effects on speech naturalness following intervention were assessed by entering the mean naturalness rating for the two speaking tasks into a mixed model ANOVA with group as the between-subjects factor (tDCS, sham) and time point as the within-subjects factor (baseline, 1 week, 6 weeks). An independent samples *t*-test was used to test change in Beck Anxiety Inventory scores between the two groups, 6 weeks post-intervention.

The means of changes from baseline in % disfluent syllables, with 95% confidence intervals (CI), were calculated for the tDCS and sham groups separately, along with the differences in these means between the two groups. Cohen’s *d* was calculated for the effect sizes of the group differences. The change from baseline in % disfluent syllables was also calculated as a percentage of the median % disfluent syllables at baseline to estimate the size of the change relative to the baseline rate of disfluency.

## Results

Between October 2014 and February 2016, 71 male adults who stutter were assessed for eligibility for the study. Thirty-four were ineligible either because their stuttering severity was assessed as mild (*n* = 28), which was below our cut-off of moderate severity, or because they had an additional language disorder (*n* = 2), or contra-indications to brain stimulation (*n* = 4). Seven declined to participate. Thus, 30 participants met the eligibility criteria and were recruited. All participants completed the intervention and post-intervention sessions, and were included in all the analyses. The 1-week post-intervention session was carried out on average 8 days after intervention (range 6–13 days) and the 6-week session at 40 days after intervention (range 32–53 days). Table 1 shows baseline characteristics, which were well matched between the tDCS and sham groups.

**Table 1:** Baseline characteristics

	TDCS (*n = *15)	Sham (*n = *15)
	Mean (SD)	Mean (SD)
Age at intervention (years)	34.22 (8.04)	33.25 (8.76)

	**Median (IQR)**	**Median (IQR)**

% Disfluent syllables	11.97 (9.04)	12.87 (6.26)
SSI-4	27.00 (9.00)	27.00 (5.50)
OASES	3.00 (0.41)	2.84 (0.75)
BAI	11.00 (22.00)	7.00 (12.00)
Speech naturalness	5.50 (2.75)	5.00 (3.00)

SSI-4 scores range from 0 to 56, with higher scores indicating greater severity; OASES scores are ratings and range from 0 to 5, with higher scores indicating greater negative impact; Beck Anxiety Inventory (BAI) scores range from 0 to 63, with higher scores indicating more severe anxiety. Speech naturalness ratings ranged from 1 to 9, with 1 being highly natural sounding speech. IQR = interquartile range.

One participant in the tDCS group was an extreme statistical outlier [>3 standard deviations (SD) from the group mean] with regard to baseline stuttering, but this participant’s change from baseline scores were not outliers. Data from all participants were included in the primary analysis, according to the intention-to-treat principle. However, we also completed sensitivity analyses (Thabane *et al.*, 2013) by re-running all analyses excluding the participant with outlying baseline scores, to evaluate the robustness of the treatment effect. The sensitivity analyses resulted in minimal change to the tDCS group mean, and did not alter the pattern of results regarding the effects of tDCS.

**Figure 3:**
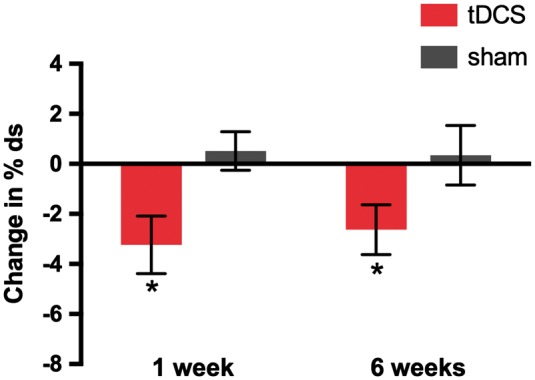
**Effect of tDCS on the primary outcome measure: change from baseline in speech disfluency. **Bars indicate mean change from baseline in % disfluent syllables (% ds) measured at 1- and 6-weeks post-intervention averaged across speech samples obtained during reading and conversation. Red = tDCS group; grey = Sham group. Error bars indicate standard error of the mean (SEM). Asterisks mark the significant main effect of tDCS.

**Figure 4:**
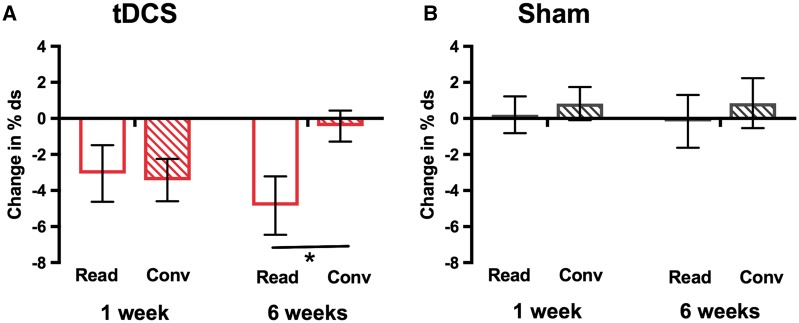
**Effect of tDCS on reading and conversation tasks separately.** Bars indicate mean change from baseline in % disfluent syllables (% ds) measured at 1- and 6-weeks post-intervention for the two speaking tasks in the (**A**) tDCS and (**B**) Sham groups. Unfilled bars = reading task (Read), striped bars = conversation task (Conv). Error bars indicate SEM. There was a significant interaction between time point and task for the tDCS group only (the significant task difference at 6 weeks post-intervention is marked with an asterisk).

Table 2 shows mean change and confidence intervals per group for all outcome measures. SSI-4 and OASES subscores are also included, for completeness. Figure 3 shows mean change in % disfluent syllables, the primary outcome measure, for both groups, at both post-intervention time points. Figure 4 shows the changes from baseline disfluency for the two speaking tasks in each group separately.

**Table 2:** Summary of mean changes from baseline per group for the primary and secondary outcomes

	TDCS (*n* = 15)		Sham (*n* = 15)	
	Mean at 1 week (95% CI)	Mean at 6 weeks (95% CI)	Mean at 1 week (95% CI)	Mean at 6 weeks (95% CI)
% Disfluent syllables	−3.24 (−5.24 to −1.24)	−2.63 (−4.87 to −0.39)	0.51 (−1.48 to 2.51)	0.34 (−1.89 to 2.58)
**SSI-4 total**	−7.13 (−9.60 to −4.66)	−3.40 (−5.36 to −1.44)	−2.27 (−4.74 to 0.20)	−1.53 (−3.49 to 0.42)
Frequency	−3.40 (−4.89 to −1.90)	−0.93 (−1.76 to −0.11)	−1.60 (−3.16 to −0.04)	−0.07 (−1.31 to 1.18)
Duration	−2.67 (−3.97 to −1.37)	−1.60 (−2.46 to −0.74)	−1.87 (−2.93 to −0.80)	−1.60 (−2.80 to −0.40)
Physical concomitants	−1.07 (−2.54 to 0.41)	−0.87 (−2.10 to 0.34)	1.20 (−0.17 to 2.23)	0.20 (−0.66 to 1.07)
**OASES total**	n/a	−0.23 (−0.44 to −0.01)	n/a	−0.13 (−0.27 to 0.02)
General information	n/a	−0.20 (−0.14 to 0.01)	n/a	−0.16 (−0.28 to −0.03)
Reactions to stuttering	n/a	−0.32 (−0.61 to −0.33)	n/a	−0.13 (−0.23 to −0.03)
Communication in daily situations	n/a	−0.16 (−0.44 to 0.11)	n/a	−0.07 (−0.26 to 0.12)
Quality of life	n/a	−0.20 (−0.34 to 0.03)	n/a	−0.15 (−0.38 to 0.07)

Subscores on the SSI-4 and OASES are included for completeness. n/a = not assessed.

For our primary outcome measure, change from baseline in % disfluent syllables across both tasks, we found significantly greater reduction in disfluency in the tDCS group relative to the sham group who showed minimal change from baseline [main effect of group, *F*(1,28) = 7.21, *P = *0.012, Cohen’s *d* = 0.98; Fig. 3]. Across the two groups, the change in % disfluent syllables did not significantly differ between the two post-intervention time points (1 and 6 weeks) or between the two speech tasks (reading and conversation) (no significant main effects of task: *P = *0.144; or time point: *P = *0.774). However, in the same ANOVA there were significant interactions between task and time point [*F*(1,28) = 6.62, *P = *0.016] and among task, time point and group [*F*(1,28) = 4.77, *P = *0.037]. The three-way interaction was examined using repeated-measures ANOVA for each group separately with factors of task and time point. For the tDCS group, there was a significant interaction between task and time point [*F*(1,14) = 11.13, *P = *0.005; Fig. 4A] and this was not significant for the sham group (*F* < 1, *P = *0.786; Fig. 4B). Examination of the means in Fig. 4A suggests that the task × time point interaction in the tDCS group is due to maintenance of the reduced disfluency relative to baseline for the reading task at 6 weeks but a return to baseline disfluency levels for the conversation task (effect of task, *P = *0.020).

The change in fluency from baseline across speaking tasks (the primary outcome of the trial) was −3.24% disfluent syllables at 1 week and −2.63% disfluent syllables at 6 weeks after intervention, for the tDCS group. This change expressed as percentage of the baseline % disfluent syllables (11.97%), represents a 27% reduction in disfluency at 1 week and 22% at 6 weeks. In contrast, the change from baseline in % disfluent syllables for the sham group represented a 4% increase in disfluency at 1 week and a 3% increase at 6 weeks (percentage of their baseline of 12.87%).

**Figure 5:**
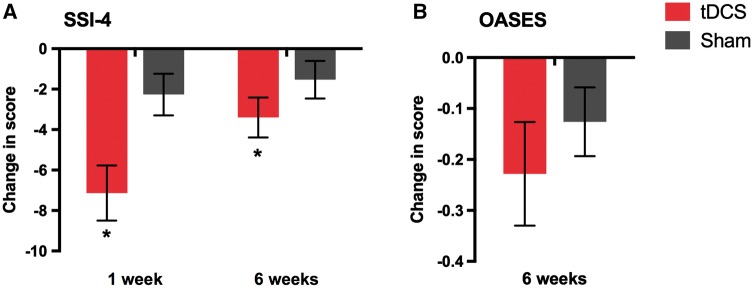
**Effects of tDCS on secondary outcomes: change from baseline in SSI and OASES scores.** Bars indicate mean change from baseline in (**A**) SSI-4 scores at 1- and 6-weeks post-intervention, and (**B**) OASES scores at 6-weeks post-intervention, for the tDCS (red) and sham (grey) groups. Error bars indicate SEM. Asterisks mark the significant main effect of tDCS on the SSI-4 change scores. There was no significant difference between groups for the OASES.

For our secondary outcome measure of stuttering severity, we found a significantly greater reduction in SSI-4 score in the tDCS relative to the sham group [main effect of group, *F*(1,28) = 6.31, *P = *0.018; Cohen’s *d* = 0.92; Fig. 5A]. The reduction in SSI-4 was significantly larger at 1 week compared with 6 weeks post-intervention, for both groups [significant main effect of time point, *F*(1,28) = 8.73, *P = *0.006; Fig. 5A]. The interaction between group and time point was not significant [*F*(1,28) = 3.94, *P = *0.057].

For our other secondary outcome measure, change from baseline in the OASES, which was measured only at 6 weeks post-intervention, we found no significant effect of tDCS [independent samples *t*-test, *t*(28) = −0.84, *P = *0.410; Cohen’s *d* = 0.31; Fig. 5B]. Examination of the means shown in Fig. 5B reveals that the OASES scores were reduced relative to baseline in both groups.

**Figure 6:**
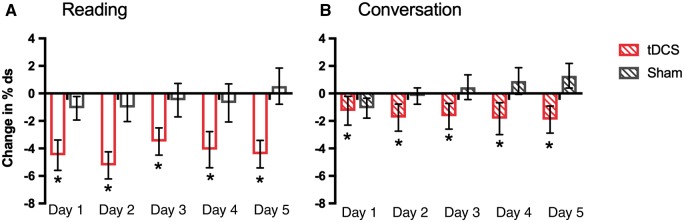
**Effects of tDCS on speech disfluency during the 5-day intervention.** Bars indicate mean change in % disfluent syllables (% ds) from baseline in speech sample during (**A**) reading (open bars) and (**B**) conversation (striped bars) tasks on Days 1 to 5 during the intervention for the tDCS (red) and sham (grey) groups. Error bars indicate SEM. The significant main effect of stimulation is marked with asterisks.

In separate exploratory analyses, we examined the effects of tDCS on change from baseline in % disfluent syllables during the 5-day intervention. There was a significantly larger reduction in % disfluent syllables over the 5 days of the intervention in the tDCS relative to the sham group [main effect of group, *F*(1,28) = 9.53, *P = *0.005, Cohen’s *d* = 1.13; Fig. 6]. This main effect did not interact with speech task but in both groups the change from baseline in % disfluent syllables was significantly greater for the reading compared with the conversation task [main effect of task, *F*(1,28) = 5.36, *P = *0.028]. Although the interaction was not significant, it is worth noting that the difference between reading and conversation is clearly evident in the tDCS group and minimal in the sham group (Fig. 6). There was no main effect of time (i.e. day of intervention) nor interaction involving time, task or group.

There were no serious adverse effects during the trial. TDCS adverse effects were limited to the mild symptoms commonly reported in previous studies (e.g. itching and tingling under the electrodes). Adverse effects significantly reduced over the course of the intervention for both groups [main effect of day: *F*(4,104) = 2.79, *P* = 0.030], but did not significantly differ between the tDCS and sham groups (no significant effect of stimulation group, or interaction with intervention day). Neither tDCS nor the behavioural intervention alone affected speech naturalness (no significant main effect of stimulation group, time point or interaction). There was no significant difference between the groups in the change in Beck Anxiety Inventory scores, following intervention.

## Discussion

This first randomized controlled trial using tDCS to treat developmental stuttering showed that tDCS in combination with a behavioural fluency intervention significantly enhanced speech fluency compared with sham stimulation. Furthermore, this benefit remained evident at least 6 weeks post-intervention. For the primary outcome measure, the percentage of disfluent syllables averaged across reading and conversation tasks at 1 and 6 weeks post-intervention was significantly reduced in the tDCS group relative to the sham group. Similarly, the combination of tDCS and fluency intervention significantly reduced scores on a standardized measure of stuttering severity, SSI-4, relative to sham stimulation. This effect also persisted for 6 weeks post-intervention. The magnitude and the persistence of improvements for the tDCS group in these outcomes indicate the clinical potential for tDCS as an adjunctive therapy.

The SSI-4 was included in the trial as a widely recognized standardized clinical measure, which provided complementary information to the primary outcome measure regarding fluency disruptions. Specifically, % disfluent syllables is a highly sensitive measure of stuttering frequency, whereas the SSI-4 sacrifices some sensitivity (by conversion to scaled scores), but incorporates important information regarding duration of stuttered moments, and of concomitant features, such as tic-like facial or body movements. The effects of tDCS measured by SSI-4 were consistent with those reported for the primary outcome: this composite measure of stuttering symptoms was significantly reduced relative to sham across both post-intervention time points. The subscores of the SSI-4 were not statistically analysed separately. However, inspection of the means showed that the size of reductions was larger in the tDCS group than the sham group for all subscores (frequency, physical concomitants and duration), except for the duration subscore at 6 weeks post-intervention, when the group means did not differ (see Table 2).

No significant benefit of tDCS was found for the OASES self-assessment, our other secondary outcome measure. However, both groups showed some reduction in the negative psycho-social impact of stuttering following intervention. We included the OASES as a measure of psycho-social impact; however, the assessment has a broader scope, encompassing all domains of health and disability within the World Health Organisation ICF framework (http://www.who.int/classifications/icf/en/). The subscores of the OASES were not separately analysed but small reductions were seen for both groups across all subscores (assessing general understanding of stuttering, reactions to stuttering, communication in daily situations and quality of life; Table 2). The small changes on OASES total score seen in both groups may have been associated with involvement in the trial, which may have increased understanding of stuttering, and increased social contact during the study, or because of the experience of a positive listener response when speaking under challenging conditions. It is perhaps unsurprising that the brief intervention used for this first randomized controlled trial in stuttering had no additional effect on the psycho-social impact of living with a stutter, as the relationship between this and stuttering severity is complex.

Our primary outcome measured change in fluency across the two speaking tasks, reading and conversation, but we were also interested in potential differences in sensitivity to tDCS between the two tasks. In the tDCS group, the significant reduction in disfluency observed 1 week post-intervention was maintained for the reading task at 6 weeks post-intervention but had decreased significantly for the conversation task (i.e. it had returned towards baseline levels). Changes in disfluency for reading and conversation were also considered separately in our additional exploratory analysis of the time-course of tDCS effects during the intervention. We found that tDCS reduced disfluency significantly across the 5 days of the intervention and that the disfluency decreases were greater for the reading than conversation tasks. It appears therefore that speech samples taken during reading tasks provide a more sensitive measure of disfluency. This may be because it is impossible to avoid difficult words or phrases (i.e. those on which stuttering is predicted) when reading text, whereas during conversation people who stutter commonly report using such avoidance strategies (Riley *et al.*, 2004). Nevertheless, fluency during conversation might be considered a more ecologically valid outcome measure of a trial aimed at improving speech fluency. The return to baseline at 6 weeks for measures of dysfluency during conversation in the tDCS group is somewhat disappointing therefore. Testing combined tDCS and behavioural therapy paradigms to induce more robust increases in fluency during conversation, for example using a longer intervention period, will be important in the ongoing development of this approach for clinical application.

We used a combination of behavioural interventions that have been shown to immediately, and relatively effortlessly, induce speech fluency in people who stutter. As predicted these interventions induced fluency in both sham and tDCS groups in the current study. Temporary fluency enhancements have been shown to be associated with normalized activity in the left inferior frontal cortex (Wu *et al.*, 1995; Fox *et al.*, 1996; Toyomura *et al.*, 2011). We propose that tDCS over the left inferior frontal cortex during the fluent mode of speaking facilitated plasticity of the frontal speech network and prolonged its normalized functioning, resulting in lasting improvements in fluency. The neural changes underlying the lasting fluency improvements need to be investigated in future studies.

The tDCS parameters used in the present study were chosen as they have previously been shown to be effective for modulating speech and motor learning (Allman *et al.*, 2016; Marangolo *et al.*, 2011). However, systematic direct comparison of various tDCS protocols on speech fluency would help to gain more information on the potential clinical benefits of this approach.

A side-effect of explicitly learning new speech patterns in fluency therapy can be a reduction in speech naturalness (Metz *et al.*, 1990; O'Brian *et al.*, 2003; Tasko *et al.*, 2007), particularly in the early stages. Reduced naturalness following therapy can result in a more negative listener response than to stuttering itself (Stuart and Kalinowski, 2004), and reduce the maintenance of therapy gains (Onslow *et al.*, 1992). The current study aimed to induce fluent speech immediately and with minimal effort, importantly, not to negatively impact speech naturalness. The maintenance of natural-sounding speech following the combination of tDCS with temporary behavioural fluency enhancement in this paradigm is noteworthy. TDCS as an adjunctive therapy for stuttering would have particular impact if maintenance, or even improvement, of speech naturalness is shown to be a replicable outcome.

The positive outcome of this trial has relevance more broadly to the application of tDCS to speech and language disorders, both acquired and developmental. Our results here are consistent with previous work in aphasia (see reviews by Holland and Crinion, 2012; Monti *et al.*, 2013; Sandars *et al.*, 2016). Of particular relevance to the current trial are two studies showing increased speech motor skill following anodal tDCS over left inferior frontal cortex (Marangolo *et al.*, 2011, 2013) in two small samples of patients with acquired apraxia of speech (three and eight patients, respectively). Our larger sample of stuttering participants adds support to the claim that applying anodal tDCS over left inferior frontal cortex can increase speech motor rehabilitation outcomes. There has been limited research using tDCS in developmental disorders of communication, perhaps because of an understandable caution regarding interacting with neuroplastic processes during childhood. However, tDCS has an interesting potential for augmenting the limited therapeutic outcomes for adults living with persistent developmental difficulties, the impact of which can be considerable (Clegg *et al.*, 2005; Craig *et al.*, 2009; Tanner, 2009). One study found that tDCS over area V5/MT combined with a 5-day course of reading therapy improved reading speed and fluency in adults with developmental dyslexia, with benefits persisting 1 week after the intervention (Heth *et al.*, 2015). To our knowledge, there are no other studies in adults with developmental disorders of communication, and none in developmental disorders of speech. Our study suggests that tDCS may be usefully applied to persistent developmental communication disorders, and may have particular value where behavioural therapies alone have failed to produce lasting positive outcomes.

In summary, we found that daily application of 20 min, of 1-mA anodal tDCS over the left inferior frontal cortex combined with tasks performed under choral and metronome-timed speaking conditions for five consecutive days improved speech fluency in 15 male adults who stutter. Another 15 adults who stutter showed no change in speech fluency from the same behavioural intervention paired with sham stimulation. These positive findings provide encouragement for future research in developmental stuttering and other disorders of speech and language. Clinical interventions could be extended to use non-invasive brain stimulation in combination with established speech therapy methods including those aimed at reducing the negative impact of living with these conditions. Brain stimulation using tDCS has moderate costs, and the devices are simple to use, requiring minimal training. Using tDCS stimulation to improve the efficacy of a therapy could reduce the number of sessions required by an individual, offering savings and allowing more individuals to be treated. Furthermore, it could improve outcomes and prevent relapses. Further work is needed, however, to investigate the limitations of this method, its underlying mechanisms, and the optimal tDCS paravmeters for increasing fluency.

## Abbreviations

OASES: Overall Assessment of the Speaker’s Experience of Stuttering
SSI-4: Stuttering Severity Instrument, version 4
tDCS: transcranial direct current stimulation
